# BTLA expression declines on B cells of the aged and is associated with low responsiveness to the trivalent influenza vaccine

**DOI:** 10.18632/oncotarget.4597

**Published:** 2015-06-23

**Authors:** Senthil Kannan, Raj K. Kurupati, Susan A. Doyle, Gordon J. Freeman, Kenneth E. Schmader, Hildegund C.J. Ertl

**Affiliations:** ^1^ Biomedical Graduate Group, University of Pennsylvania, Philadelphia, PA, USA; ^2^ The Wistar Institute, Philadelphia, PA, USA; ^3^ GRECC, Durham VA Medical Center and Center for the Study of Aging and Human, Development and Division of Geriatrics, Department of Medicine, Duke University Medical Center, Durham, NC, USA; ^4^ Dana-Farber Cancer Institute, Harvard Medical School, Boston, MA, USA

**Keywords:** BTLA, aging, HSV, immunosenescence, influenza

## Abstract

Virus-neutralizing antibody and B cell responses to influenza A viruses were measured in 35 aged and 28 middle-aged individuals following vaccination with the 2012 and 2013 trivalent inactivated influenza vaccines. Antibody responses to the vaccine strains were lower in the aged. An analysis of B cell subsets by flow cytometry with stains for immunoregulators showed that B cells of multiple subsets from the aged as compared to younger human subjects showed differences in the expression of the co-inhibitor B and T lymphocyte attenuator (BTLA). Expression of BTLA inversely correlated with age and appears to be linked to shifting the nature of the response from IgM to IgG. High BTLA expression on mature B cells was linked to higher IgG responses to the H1N1 virus. Finally, high BTLA expression on isotype switched memory B cells was linked to better preservation of virus neutralizing antibody titers and improved recall responses to vaccination given the following year.

## INTRODUCTION

In the 2012/13 Influenza season, infections with H3N2 virus were predominant. Final analyses of vaccine efficacy in the US and Canada reported an efficacy of ∼ 32% for influenza A viruses in the general population. However, in people over 65 years of age, the efficacy against influenza A viruses only reached 9% [1]. Overall, vaccine-induced protection against the most prevailing Influenza A virus was far from optimal in spite of a good match between the vaccine and the circulating strains. Poor vaccine efficacy in the elderly is of particular concern since the elderly experience increased morbidity and mortality following infection [2]. Primary B cell responses in the elderly are commonly low and short-lived, resulting in antibodies with low affinity [3]. The elderly are also prone to develop recall responses that match previously circulating strains, a phenomenon known as antigenic sin caused by prior recurrent influenza virus infections [4]. In addition, germinal center formation, which is required for induction of long-lived affinity-matured plasma and memory B cells, is decreased in the aged [5]. Some of the defects of B cell responses in the elderly are also caused by an age-related decline of helper functions from CD4^+^ T cells [6]. This phenomenon is marked by reduced expression of critical costimulatory receptors, such as CD28 and CD40 ligand, which in turn, are essential for activation of B cells and germinal center formation [7]. Additionally, similar to observations made in T cells, expression of coinhibitory receptors such as programmed death (PD)-1 or lymphocytes activation gene (LAG)-3 may increase upon aging, tipping the balance toward inhibition of immune responses [8].

To further define age-related defects in responses to the 2012/2013 TIV, we tested virus neutralizing antibody (VNA) and B cell responses in a cohort of 35 aged individuals of or above 65 years of age in comparison to 28 younger individuals between 30-40 years of age. VNA responses were tested against the two influenza A virus strains of the vaccine, i.e., H1N1 A/California/7/2009 pdm09-like virus and H3N2 A/Victoria/361/2011 virus. Immune responses were tested from blood at baseline and on days 7 and 14 after TIV. We had conducted a study for individuals vaccinated with the 2011/2012 TIV and shown that circulating B cell responses peak at these time points [9]. Cellular responses were analyzed with regard to numbers of circulating cells of different B cell subsets, i.e., transitional B cells, mature naïve B cells, unswitched and switched memory B cells, double-negative B cells, and antibody secreting cells (ASCs) defined by stains for CD19, IgD, CD20, CD27, and CD38. In addition, we characterized expression of the B and T lymphocyte attenuator (BTLA), which upon interactions with the herpes virus entry mediator (HVEM) provides inhibitory signals [10].

Our results show that, as expected, the younger individuals mounted higher VNA responses to both viruses and had higher numbers of naive B cells compared to the aged. BTLA was significantly higher expressed on B cells from younger compared to aged individuals at baseline. Expression levels of BTLA, as well as percentages of BTLA^hi^ B cells, selectively increased in the aged after vaccination so that differences between the two cohorts cease to be significant. Individuals with high BTLA on their mature B cells had better IgG responses to the H1N1 virus, compared to those with low BTLA. Further analysis showed that individuals with high BTLA expression levels showed better preservation of H1N1- and H3N2-specific VNA titers and superior booster response to the next annual dose of TIV compared to those with BTLA^lo^ switched memory B cells. Overall these results show that a decline of BTLA during immunosenescence may contribute to lack of sustained antibody responses in the aged and to a reduction in their ability to mount recall responses.

## RESULTS

### Characteristics of the vaccine recipients

To compare immune responses of aged and younger individuals to the two influenza A virus strains of 2012/2013 TIV, a total of 35 aged individuals of or above 65 years of age and 28 younger individuals between 30-40 years of age were enrolled in the Durham-Raleigh-Chapel Hill of North Carolina. Briefly, the aged were on average 76 years of age ranging from 66 to 88 years of age, with 64% females and 95% Caucasians. Most individuals but for 4 had been vaccinated in 2011, the majority (58%) reported annual vaccinations with TIV. Younger individuals were on average 35 years of age with 64% females, 75% Caucasians, 21% African Americans and 4% Asians. All but two individuals received the 2011/12 Flu vaccine and 43% reported annual vaccination to influenza. Some of these individuals were re-vaccinated the following year with the 2012/13 TIV.

### VNA responses to the influenza A virus strains of 2012/13 TIV

**Figure 1:**
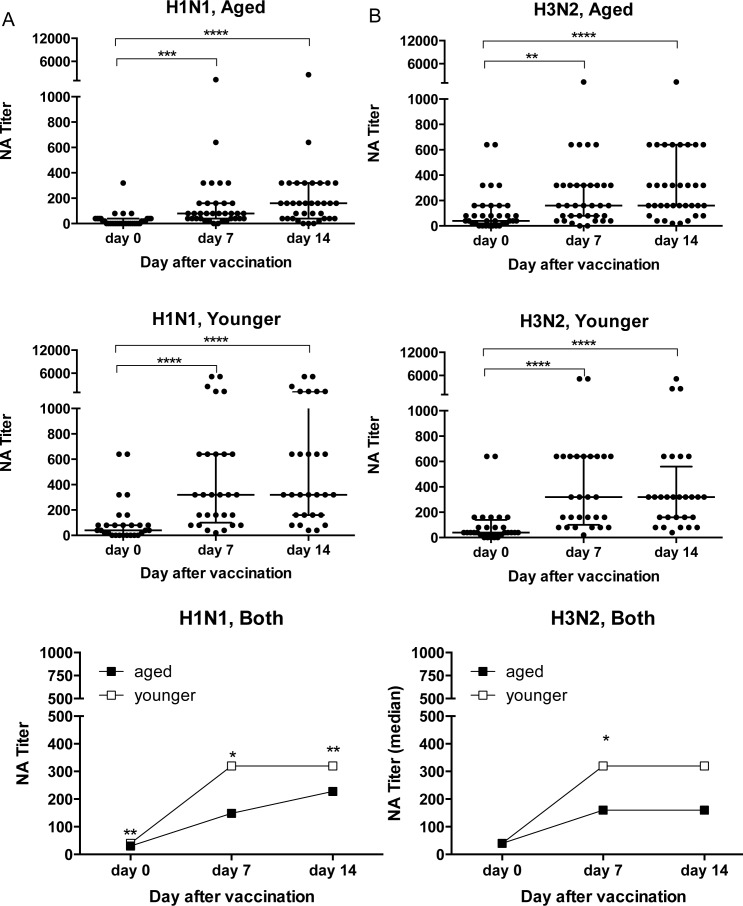
VNA Responses to Influenza A Viruses Sera were tested by a microneutralization assay on H1N1 California/7/2009 **A.** and H3N2 Victoria/361/2011 **B.** virus. Graphs on the top and in the middle show VNA responses of aged and younger individuals at baseline and on days 7 and 14 after TIV. Data are shown for individual sera, lines show medians ± Interquartile range (IQR). Graphs on the bottom show median titers of the two cohorts. (*) Indicates significant differences between titers on day 0 compared to days 7 and 14 in graphs that show individual sera calculated by Friedman test with Dunn correction with the following p - values: age, H3N2: d7 *p* = 0.0013, all other comparisons *p* < 0.0001. In the graphs showing median titers for both cohorts significant differences between the young and aged indicated by (*) were calculated by Wilcoxon matched-pairs signed rank test with the following p-values. H1N1: d0 *p* = 0.0025, d7 *p* = 0.0038, d14 *p* = 0.037.

At baseline before vaccination with the 2011/12 TIV, VNA titers to H1N1 California//7/2009 (from here on referred to as H1N1) were higher in the younger individuals (*p* = 0.0025 by Wilcoxon Rank analysis); titers to H3N2 Victoria/361/2011 (from here on referred to H3N2) were comparable (Figure 1). After vaccination, both cohorts developed increased VNA titers to H1N1 and H3N2. VNA responses to H1N1 tested at baseline or on days 7 and 14 after vaccination were significantly higher in younger individuals (Figure 1A). Responses to H3N2 virus were also higher in the younger cohort, although this only reached significance for day 7 (Figure 1B).

### Changes in circulating B cells

**Figure 2:**
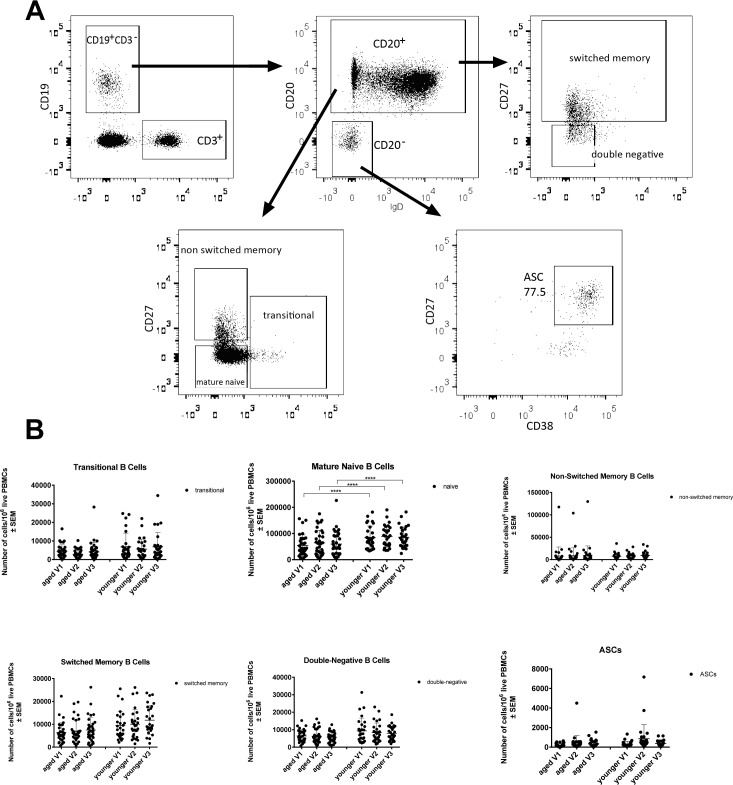
**Numbers of circulating B cell subsets: Graphs show the different B cell subsets, i.e.,** transitional B cells (CD19^+^CD20^+^IgD^+^CD27^+/−^CD38^+/−^), mature naïve B cells (CD19^+^CD20^+^IgD^+^CD27^−^CD38^−^), non-switched memory B cells (CD19^+^CD20^+^IgD^+^CD27^+^CD38^−^), switched memory B cells (CD19^+^CD20^+/−^IgD^−^CD27^+^CD38^−^), double-negative B cells (CD19^+^CD20^+^IgD^−^CD27^−^CD38^−^) and antibody secreting cells. **A.** shows the gating scheme after gating on live single lymphoid cells. **B.** shows cell counts normalized to 10^6^ live PBMCs, with error bars indicating Standard Error of the Mean (SEM). (****) indicates *p*-values < 0.0001 as calculated by two-way ANOVA, corrected for multiple comparisons with Tukey.

Numbers of cells belonging to different B cell subsets were tested for by flow cytometry upon staining of PBMCs with antibodies to lineage defining markers (Figure 2). Numbers of naïve B cells (CD19^+^IgD^+^) in blood were at all three time points higher in the younger individuals (*p* < 0.0001). There was a trend towards increased numbers of transitional B cells, which form a link between immature B cells in bone marrow and mature naïve B cells in the periphery in younger individuals; this failed to reach significance. The same was seen for double-negative IgD^−^CD27^−^ B cells, which may reflect exhausted B cells that have previously been described to be more common in the aged [11], as well as for switched memory B cells. Numbers of cells within the individual subsets were primarily stable over time, but ASCs in both age groups individuals showed a non-significant trend towards increases on day 7 as compared to baseline.

### Age-related differences in BTLA expression on B cells

**Figure 3:**
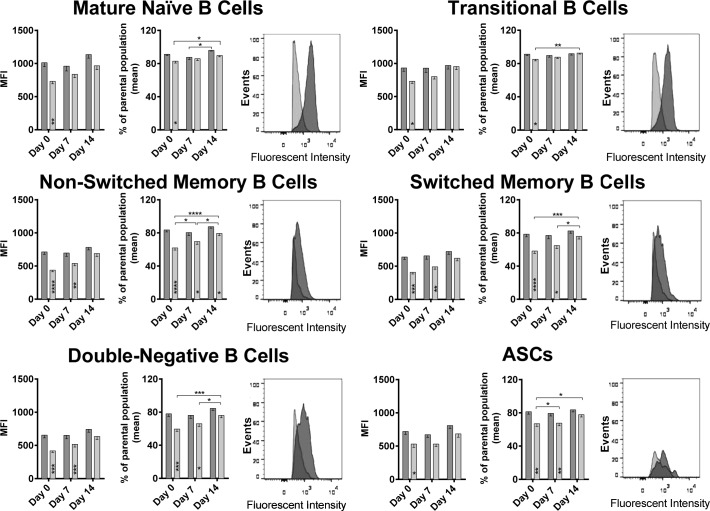
BTLA expression on B cell subsets: Mean Fluorescent Intensity (MFI) of BTLA on various B cell subsets at all three time points tested, are shown Darker bars/histograms represent the younger cohort, and the lighter bars/histograms represent the aged cohort. Histograms of a representative pair show the MFI intensity on the x-axis and events normalized to Mode on the y axis. Within each cell subset, the first graph shows the MFI with error bars denoting Standard Error of Mean (SEM). The second graph shows the % of cells that were BTLA^hi^ over the parental population. (*) within the bars indicate statistically significant differences between the two age groups at that time point. (*) above the bars indicate statistically significant differences within the same age group, between different time points as indicated by the lines. P-values were calculated using two way ANOVA, corrected for multiple comparisons using the Holm-Sidak correction.

**Figure 4:**
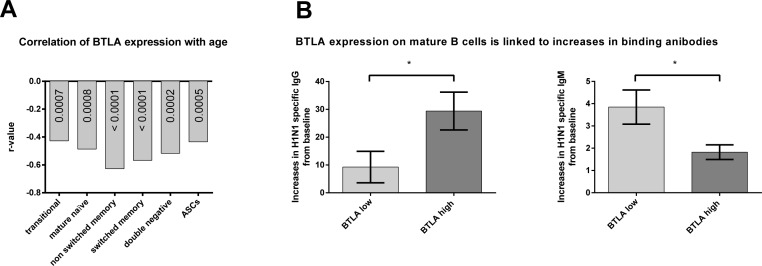
Correlation of BTLA expression with age and BTLA related increases in antibody titers The graph in **A.** shows the r-values of correlation of BTLA Mean Fluorescent Intensity (MFI) with age, as calculated by nonparametric Spearman correlation. All *p*-values were highly significant (< 0.00001). Mature naïve: *p* = 0.00008; Non Switched Memory: *p* = 7.89e^−7^; Transitional: *p* = 0.00066; Double negative: *p* = 0.00002; Switched memory: *p* = 2.21e^−6^; ASC: *p* = 0.00053. The graphs in **B.** show the mean (±SEM) increases in antibody titers to the H1N1 influenza virus, in individuals with either high BTLA on their mature B cells (BTLA high) shown in dark grey or low BTLA on their mature B cells (BTLA low) shown in light grey. (*) indicates statistical significance (*p* < 0.05) as calculated using the Mann Whitney test. *p* = 0.028 for IgG. *p* = 0.035 for IgM. Antibody titers were measured by ELISA.

It has been shown previously for T cells that the expression of co-inhibitors changes upon aging [8]. We tested B cells for a number of different markers at baseline and upon vaccination, BTLA was found to show significant age-related differences (Figure 3). At baseline, BTLA expression and percentages of BTLA^hi^ cells were significantly higher for B cells of all subsets from younger than aged individuals. After vaccination, expression levels slightly increased on aged B cells while percentages of BTLA^hi^ cells increased significantly in the aged for several of the B cell subsets. By day 7, differences between aged and younger B cells were only significant for non-switched memory B cells, switched memory B cells and double-negative B cells. By day 14 after vaccination, both levels of BTLA expression and percentages of BTLA^hi^ cells became comparable for all B cell subsets between the two age groups. BTLA expression at baseline showed a strong negative correlation with the biological age of the individuals on all B cell subsets again supporting that expression of the co-inhibitor declines upon aging (Figure 4A).

### BTLA expression on mature B cells indicates higher increases in antibody titers

To see if BTLA expression was related to antibody production, we looked at antibody titers of individuals with either high or low levels of BTLA on their mature B cells, irrespective of age. We selected ten individuals who had either the highest (BTLA high) or lowest (BTLA low) expression on their mature B cells, at baseline/on the day of vaccination, and looked at their virus specific antibody titers by ELISA, at baseline and two weeks after vaccination. Individuals with high BTLA on their mature B cells, showed the highest increase in IgG antibodies to the H1N1 virus, compared to those with low BTLA on their mature B cells (Figure 4B). This trend was reversed for IgM titers, indicating a possible role for BTLA in class switching of antibody responses.

### B cells express higher levels of BTLA than follicular T helper cells

**Figure 5:**
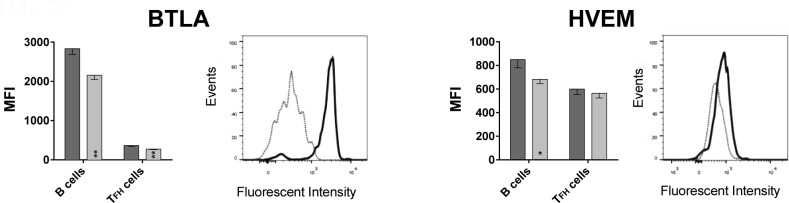
BTLA and HVEM expression on B cells and T_FH_ cells Graphs show the MFI of BTLA and HVEM on CD19^+^ B cells and CD3^+^CD4^+^CXCR5^+^PD-1^+^ T_FH_ cells, with error bars showing SEM. Dark grey bars show data from younger subjects, and light grey bars aged subjects. (*) within bars, indicate statistical significance (*p* < 0.05) between the young and aged cohorts, within that cell subset. *P*-values were calculated using multiple t-tests, corrected for multiple comparisons with Holm-Sidak correction. On B cells: *p* = 0.002; BTLA on T_FH_ cells: *p* = 0.001; HVEM on B cells: *p* = 0.023. Histograms show BTLA or HVEM expression on B and T_FH_ cells of a representative young subject. The solid black line histogram shows the respective MFI on B cells and the dotted grey line histogram shows T_FH_ cells. HVEM MFI. Both histograms show intensity on x-axis and Events normalized to Mode on the y-axis.

To formally test if BTLA signaling affects induction of ASCs, we tested if HVEM, the binding partner of BTLA, is expressed on follicular T helper (T_FH_) cells. T_FH_ cells interact with B cells during their differentiations into ASCs in lymph node follicles through interactions between PD-1 and PD-L2 [12] or ICOS and ICOS-ligand [13] expressed on T_FH_ and B cells respectively. T_FH_ cells, which generally reside in lymph nodes, can be detected at low frequencies in blood [14], were identified by positive staining for CD4, CXCR5 and PD-1. B cells were identified by staining for CD19. A stain for BTLA was included in the experiment, which used PBMC samples from 13 aged and 6 younger individuals that were not part of the original cohort shown in Figures 1, 2, 3 4. As shown in Figure 5, BTLA was expressed at high levels on B cells while T_FH_ cells were BTLA^lo^. Results from this new cohort again confirmed higher BTLA expression on B cells from younger individuals. This was also seen for T_FH_ cells. HVEM was expressed at approximately equal levels on T_FH_ cells between the younger and the aged individuals, while B cells from younger individual expressed slightly higher levels.

### High BTLA expression on memory B cells influences VNA responses

**Figure 6:**
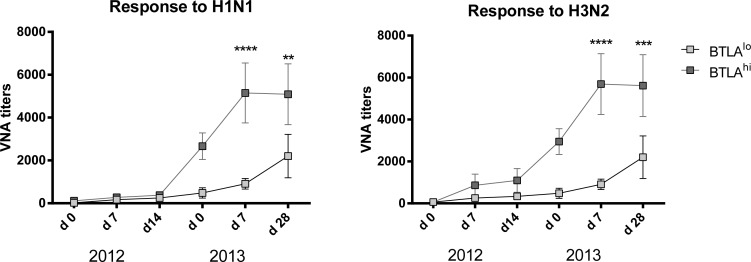
Correlation of BTLA expression and VNA responses Graphs shows VNA titers over time of selected patients with either BTLA^hi^ or BTLA^lo^ switched memory cells (as determined at baseline 2012). The x-axis shows the dates of vaccination and follow-up over the two consecutive years of vaccination. Statistically significant differences are indicated by (*) with the following p-values: Response to H1N1 2013 d7: *p* = 2.55e^−5^; d28: *p* = 0.003; Response to H3N2 2013 d7: *p* = 6.025e^−6^; d28: *p* = 0.0009.

Many of the individuals that we vaccinated in the 2012/13 season were vaccinated again by our team in the 2013/14 season. This gave us the opportunity to follow the evolution of antibody titers after an additional boost. To assess if levels of BTLA expression influenced VNA responses to the two influenza A virus strains of the vaccine we selected 10 individuals (regardless of age) that at baseline in 2012 had the highest (4 aged, 6 younger) or lowest (9 aged, 1 younger) levels of BTLA expression on their switched memory B cells. We analyzed their VNA titers on days 0, 7 and 14 after vaccination with 2012/13 TIV and then again 1 year later on day 0, 7 and 28 with 2013/14 TIV. Increases in VNA titers to the influenza A virus strains of TIV in 2012 were similar in individuals with BTLA^hi^ or BTLA^lo^ switched memory B cells although there was an insignificant trend towards higher responses of the former to H3N2 virus. More impressive was that individuals with BTLA^hi^ switched memory B cells maintained higher titers of H1N1 and H3N2-specific VNAs for the one year period between their seasonal Flu shots (compare d0 levels in 2013) and in addition mounted more robust recall responses in the following season compared to those that had BTLA^lo^ switched memory B cells at baseline in 2012 (Figure 6).

## DISCUSSION

Influenza vaccination is highly recommended for the elderly although vaccine efficacy tends to be less than optimal in this age group. Numerous studies have addressed the effect of immunosenescence on innate and adaptive immune responses in animals as well as humans [5-8, 15-23]. Human immune responses to influenza virus antigens are unique and cannot readily be recapitulated in animal models as humans are repeatedly exposed to antigens of influenza virus through infections or vaccinations. In adults, influenza vaccines elicit both recall responses to epitopes that are shared with previously encountered viruses and primary responses from naïve B and T cells to novel drifted epitopes or shifted antigens. Recall responses are expected to be particularly predominant in the elderly with their more numerous exposures to influenza viruses or vaccines, resulting in responses that can be poorly fitted to the recall antigen [4]. It has been previously shown for T cells that immunosenescence selectively affects primary responses, while memory responses elicited before aging do not become impaired [23]. This suggests that immune responses to conserved influenza antigens might remain intact even in the aged. As has also been shown for T cells, repeated recall of immune responses not only increases the pool of memory cells but also changes their activation status [24], which again may disproportionally affect the aged with their enhanced memory to influenza virus antigens.

We undertook this study with cohorts of aged and middle-aged individuals to assess age-related defects in antibody and B cell responses to TIV. A cohort of middle-aged individuals was chosen for comparison to the elderly with the expectation that these subjects had previous although less numerous exposures to influenza viruses or vaccines. Antibody responses were analyzed to identify non-responders by neutralization assays. As expected, antibody responses of the aged tended to be lower than those of younger individuals.

Our main emphasis was to identify differences in B cell subsets at baseline and upon immunization between aged and younger individuals and to assess if and how the differences may explain low responsiveness of the aged. Confirming previous results, numbers of circulating naïve B cells were lower in the aged [25] while numbers of some of the other subsets only showed a trend towards an age-related decline. Our main finding was that on all B cell subsets expression of BTLA prior to vaccination was significantly lower in the aged.

HVEM, the binding partner of BTLA also binds CD160, LIGHT, and lymphotoxin A [26]. BTLA and CD160 bind to overlapping sites while LIGHT and lymphotoxin B bind to a different domain. CD160 and BTLA provide inhibitory signals while LIGHT and lymphotoxin A act as co-stimulators. In case of simultaneous binding of an inhibitor and an activator, negative signaling prevails. The BTLA signaling pathway has mainly been studied for T cell responses where its blockade was shown to increase T cell responses to vaccines [27] and resistance to infections [28]. Negative signaling through BTLA seems to be conserved in CD8^+^ T cells of aged mice as gD-mediated blockade markedly improves their response to vaccination [29]. Downstream events of BTLA, a member of the CD28 family, remain controversial. The cytoplasmic tail of BTLA similar to that of the well characterized PD-1 immunoregulator carries a membrane proximal immunoreceptor tyrosine-based inhibition motif (ITIM) and a membrane distal immunoreceptor tyrosine-based switch motif (ITSM). These motifs, through recruitment of the Src homology (SH) proteins (SHP)-1, and SHP -2, could inhibit the activating effects of CD3/CD28 ligation leading to reduced production of cytokines and cell proliferation [30]. In addition BTLA, like CD28, contains a sequence in its cytoplasmic domain that may interact with the Grb-2 adaptor protein. This sequence could also enable it to interact either directly or indirectly with the p85 subunit of PI3 kinase, which in turn could provide survival signals [31]. Although BTLA is generally viewed as a co-inhibitor, one report showed that this may depend on the strength of antigenic stimulation; specifically, in the setting of weak stimulation by alloantigens, BTLA was found to be inhibitory while upon potent alloantigen stimulation BTLA appeared to promote the T cell response [31]. Other data showed that lack of BTLA reduced rather than augmented pathology in a colitis model [32] and promoted survival of antigen-specific CD8^+^ T cells induced by bacterial infection [33] again suggesting that BTLA may have disparate role in maintaining homeostasis of the immune system.

One interesting observation was the relationship between BTLA expression on mature B cells prior to vaccination, and increases in H1N1 specific IgG antibody titers with concomitant decreases in IgM titers. This seems to indicate that high BTLA expression on mature B cells might play a role in augmenting IgG production, specifically in shifting the response from IgM to IgG. The fact that we only saw this for the H1N1 virus, and not the H3N2 virus needs to be noted. It may reflect that H1N1 underwent a dramatic change in 2009 and is thus a fairly recent virus while variants of H3N2 have been circulating since 1968.

More intriguing was that high expression of BTLA on switched memory B cells, the main subset that responds to vaccination against influenza virus to which adult humans in general have immunological memory was linked to better preservation of VNA responses to the two influenza A virus strains of TIV and to higher recall responses upon the next dose of TIV given one year later. This in turn suggests that BTLA may promote rather than suppress the induction of long-lived plasma cells and memory B cells, a hypothesis that remains to be investigated further.

In summary our results show a decline in BTLA expression on B cells upon immunosenescence that may be linked to decreased induction of long-lived plasma cells, memory B cells, and antibody titers in the aged.

## MATERIALS AND METHODS

### Virus strains

The two influenza A vaccine strains of the 2012/13 seasonal influenza vaccine, A/California/7/2009 (H1N1) pdm09-like virus and A/Victoria/361/2011 (H3N2)-like virus were obtained from the Center for Disease Control, Atlanta, Georgia. Viruses were expanded by injecting 100 μl of 4 hemagglutinating units (HAU) of virus/ml into 10 day-old specific pathogen-free embryonated eggs, which were then incubated for 48 hrs at 35°C. After 48 hrs, the eggs were chilled at 4°C overnight. The allantoic fluids from the infected eggs were harvested and concentrated by centrifuging at 20,000rpm for 1 hr at 4°C. The concentrated viral pellet was resuspended in PBS and further purified by fractionation over 10-55% sucrose density gradients at 25,000rpm for 2 hrs. Purified viruses were serially diluted on Madin-Darby Canine Kidney (MDCK) cells to determine mean tissue culture infective dose (TCID_50_) after 3 days of infection by screening for cytopathic effects (CPE).

### Human subjects

Blood was collected after informed consent from community dwelling persons in the Durham-Raleigh-Chapel Hill area of North Carolina. Younger individuals were 30-40 years of age; older individuals were > 65 years of age. The following subjects were excluded from the study: (1) humans with immunosuppression resulting from diseases (e.g., clinically active malignancy, HIV/AIDS, immune disorders) or drugs (e.g., cancer chemotherapy, corticosteroid use); (2) individuals with significant underlying diseases that would be expected to prevent completion of the study; (3) subjects, which were bed-ridden or homebound or had intercurrent illnesses that might interfere with interpretation of study (e.g., urinary tract infection, respiratory tract infection); (4) individuals that were unlikely to adhere to protocol follow-up; (5) subjects that were involved in a conflicting study; (6) subjects that had a history of alcohol or substance abuse; (7) subjects with contraindication for influenza vaccination such as anaphylactic hypersensitivity to eggs or to other components of the influenza vaccine, and moderate or severe acute illness with or without fever, and Guillain-Barre Syndrome within 6 weeks following a previous dose of influenza vaccine. Persons with moderate to severe acute febrile illness were not vaccinated until their symptoms had abated.

From enrolled subjects demographic data and medical history including medical diagnoses, medications, vaccination to influenza and other infectious diseases, and history of influenza or influenza-like diseases during the last 5 years were recorded. Subjects were bled and then vaccinated with TIV via the intramuscular route in the deltoid muscle. Subjects were bled again on days 7 and 14 following injection of TIV. Some of the subjects were revaccinated with TIV the following years using the same procedures.

### Collection of blood and isolation of PBMCs and plasma

Blood was collected into heparinized tubes and shipped overnight to Philadelphia, PA. A 1.5 ml aliquot of each sample was set aside for serum collection. PBMCs were isolated from the remaining samples using established protocols. Specifically, blood was overlaid onto Ficoll-Paque Plus (GE Healthcare Biosciences, Piscataway Township, NJ) and spun for 30 minutes at 2000 rpm, with brake off, and at 50% acceleration. The PBMC layer at the Ficoll interface was then collected and washed twice with Hank's Balanced Salt Solution (Gibco, Grand Island, NY), by centrifuging at 2000 rpm. The washed, pelleted cells were treated with 10 ml of red blood cell lysis buffer (eBioscience, San Diego, CA). Lysis was stopped by adding 5 ml of Roswell Park Memorial Institute (RPMI) 1640 medium supplemented with 10% fetal bovine serum [FBS]. Cells were washed with Hank's Salt (HBSS). Cells were resuspended in 5ml of Dulbecco's modified Eagles medium (DMEM), live cells were counted using Trypan Blue as a diluent.

### Micro-neutralization assay

Two-fold serially diluted (1:20 to 1:10240) heat-inactivated human sera were tested for neutralizing antibodies to influenza A virus strains by micro-neutralization assays. Equal volume of 100TCID_50_ per well of the titrated virus was added to the diluted serum in 96 well plates and incubated at 37°C. After 1hr, serum-virus mixtures were added to MDCK cells that had been washed twice with serum-free Dulbecco's Modified Eagles Medium (DMEM). The cells were incubated for 2 hrs at 37°C with 5% CO_2_. The cells were washed and re-incubated with DMEM supplemented with L-1-Tosylamide-2-phenylethyl chloromethyl ketone (TPCK) trypsin for 3 days. CPEs were scored under a microscope. Neutralization titers were defined as the dilution of the serum that resulted in 50% inhibition of CPE formation.

### B cell detection by flow cytometry

Each subject sample was initially treated with Human TruStain FcX Fc Receptor Blocking solution (BioLegend, San Diego, CA) for 30 minutes, washed with PBS at 1500 rpm for 5 minutes and then stained with fluorochrome-conjugated antibodies. Samples were stained first for their extracellular markers using the following antibodies: CD19-Brilliant Violet^TM^650, CD20- Brilliant Violet^TM^570, IgD-PeCy7, CD38-PerCPCy5.5, CD3-Pacific Blue, CD14-Pacific Blue, CD27-FITC, BTLA-APC, and AmCyan Aquablue as a live cell stain. The optimal concentrations of these antibodies were determined experimentally. Samples were stained for 30 minutes at room temperature and washed with PBS. The cells were then permeabilized using Cytofix/Cytoperm (BD Biosciences) for 30 minutes at 4°C, washed with Permwash (BD Biosciences) and stained with their specific panel of intracellular antibodies. The samples were stained with the intracellular stains for 30 minutes at 4°C, washed with PBS and then resuspended in 150μl of fixative (BD Pharmingen). All antibodies were obtained from BD Biosciences (San Jose, CA) unless specified differently. The stained samples were analyzed in a LSRII flow cytometer (BD Biosciences, San Jose, CA).

### ELISA

H1N1- and H3N2-specific binding antibody isotypes were measured by ELISA. Briefly, wells of Nunc Maxisorp™ plate were coated with 10μg/ml of influenza H1N1 or H3N2 virus along with isotype standards for IgA1, IgG and IgM (Athens Research & Technology, Inc., Georgia, USA) in bicarbonate buffer overnight at 4°C. The plates were blocked with 3% BSA in PBS and incubated for 2 hrs at room temperature with heat-inactivated sera of young and aged subjects at a dilution of 1/250. The plates were washed 4X with PBS containing 0.05% tween (PBST) and incubated for 1 hr at room temperature with alkaline phosphatase conjugated mouse anti-human IgA1 at 1:1000, IgG at 1:3000 and IgM at 1:1000 dilutions (SouthernBiotech, Alabama, USA). Following the incubation, plates were washed 4X with PBST and developed using alkaline phosphatase substrate containing pNPP tablets (Sigma Aldrich, Missouri, USA) dissolved in DEA buffer. Adsorbance was recorded at 405nm. The adsorbance values were plotted against standard curves from each plate for every isotype. Antibody concentrations were determined and are expressed in μg/ml.

### Statistical analyses

Results were analyzed by multiple t-tests or 2-way ANOVA. Correlations were tested for by Spearman. P-values were corrected for multiple testing using Benjamini-Hochberg procedure. Results with corrected *p*-values < 0.05 were considered significant.
